# Supplementary material for: Enhanced Thermostability and Enzymatic Activity of cel6A Variants from Thermobifida fusca by Empirical Domain Engineering
Source: Biology (Basel). 2020 Aug 7;9(8):214. doi: 10.3390/biology9080214 (PMC7464639; doi:10.3390/biology9080214)
Supplement: Supplementary file 1 [file biology-09-00214-s001.pdf]

Table S1. Primers used for amplification of gene fragments of cel6A. [a] Restriction sites are bold and underlined.

Primer	Sequence	Restriction site [a]
P1	5'- CTA**CATATG**GCCAATGATTCCCCGTTTTACG-3'	NdeI
P2	5'- CACGCCT**AAGCTT**CCCAGTGC-3'	HindIII
P3	5'-CTT**AAGCTT**CTAGGCGATCGCCATCTCG -3'	HindIII
P4	5'- **CCATGG**CGGGCACCAACC-3'	Nco1
P5	5'- **GGATCC**GTACGTACGTCGCCGTGCAC-3'	BamH1
P6	5'- CT**GGATCC**AATGATTCTCCGTTCTAC-3'	BamH1
P7	5'- **CATATG**ACGATCGCCAACGAGTGGAAC-3'	Nde1
P8	5'- GGTCAG**CCATGG**CGCAGGTAAG-3'	Nco1
P9	5'- CTA**CCATGG**GCCAATGATTCCCCGTTTTACG-3'	Nco1

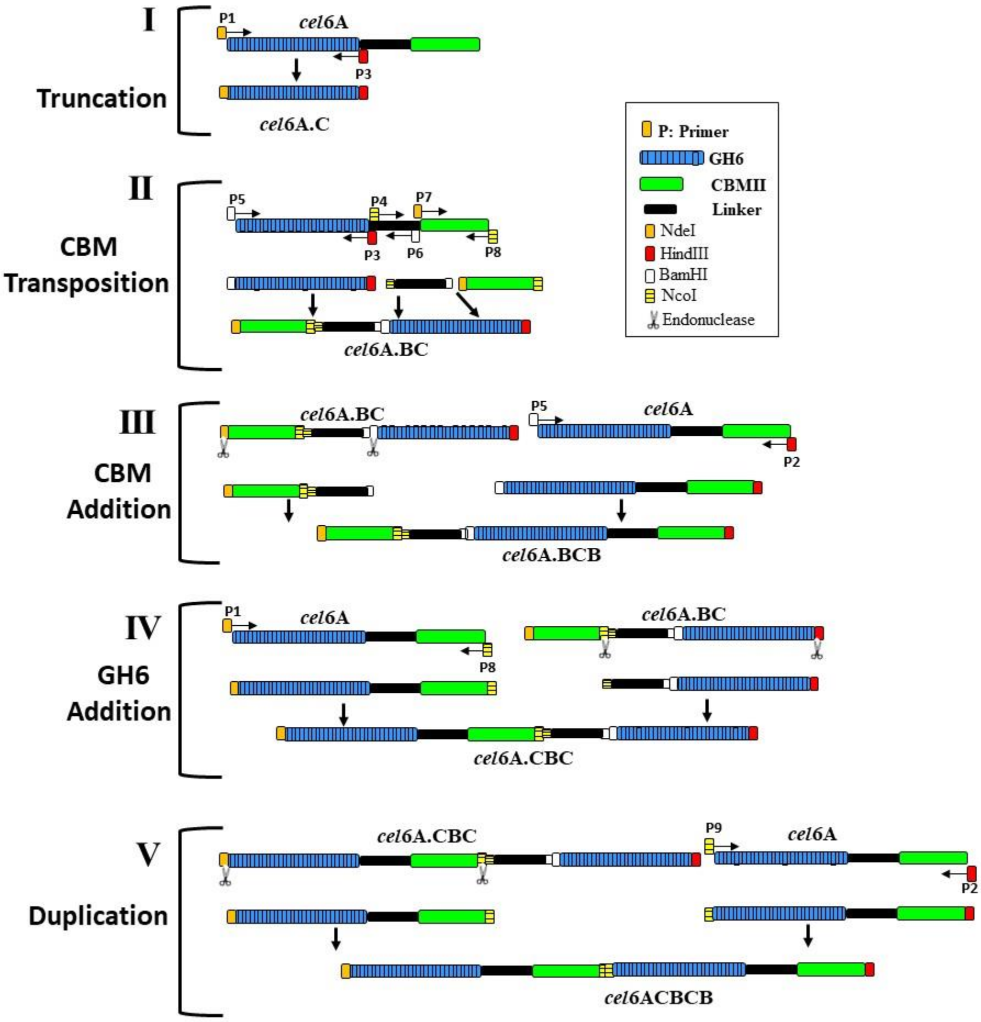

Figure S1. Schematic representation of engineering of *T. fusca* endoglucanase cel6A. (**I**) cel6A.C, (**II**) cel6A.BC, (**III**) cel6A.BCB, (**IV**) cel6A.CBC and (**V**) cel6A.CBCB.

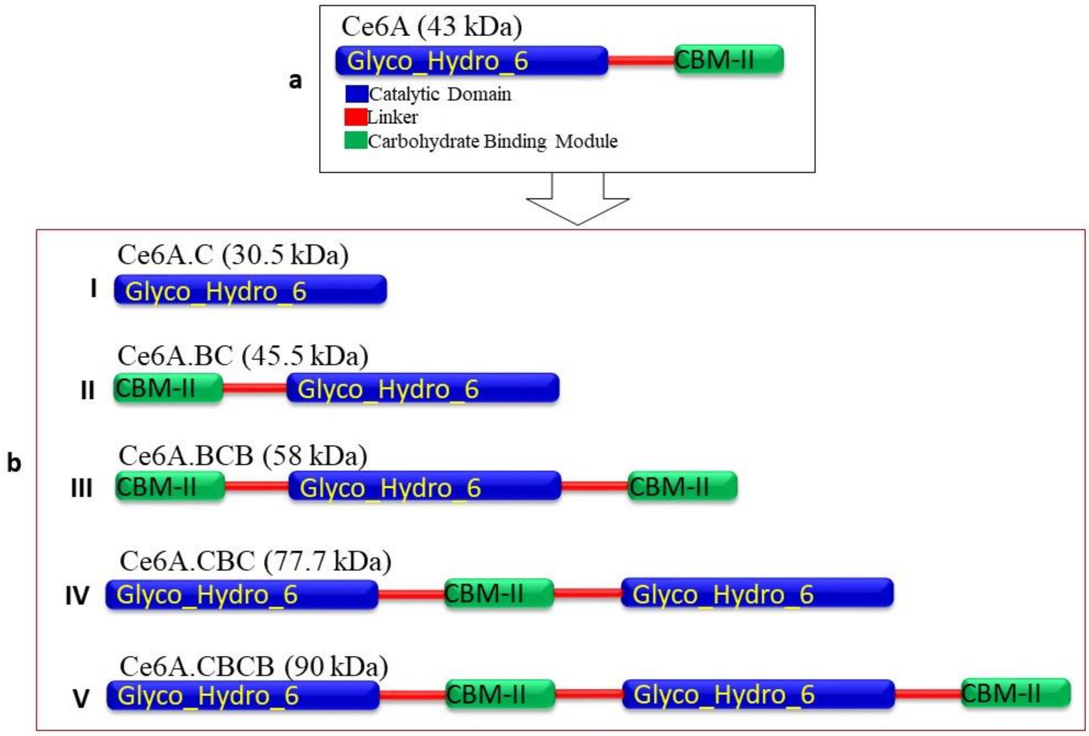

Figure S2. Schematic representation of *T. fusca* endoglucanase cel6A modular architecture, (**a**) Native cel6A, (**b**) Engineered cel6A variants; truncated (cel6A) without non catalytic domain, non-catalytic domain to N-terminus (cel6A.BC), non-catalytic domain to both N- and C- termini of catalytic domain (cel6A.BCB), catalytic domain to both N- and C- termini to the non-catalytic domain (cel6A.CBC) and duplication of native cel6A endoglucanase with their respective protein size.

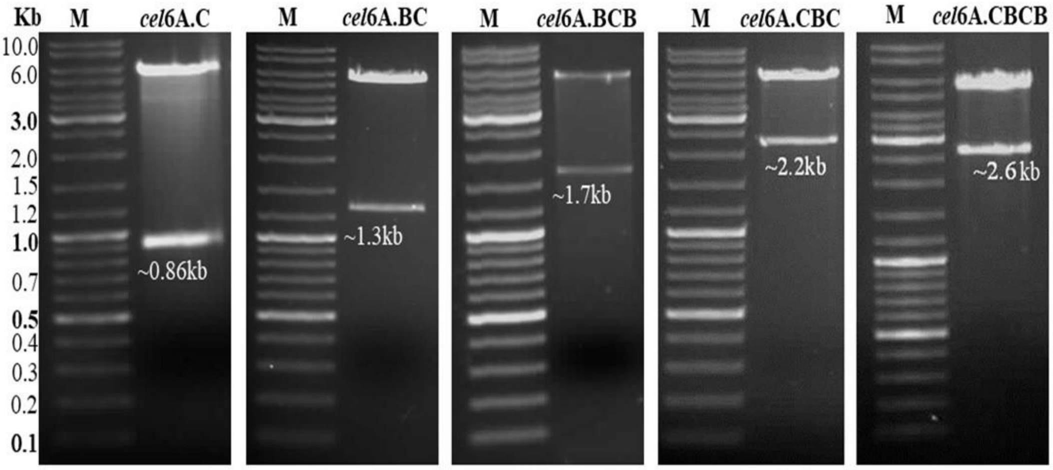

Figure S3. Agarose (0.8%) gel showing restriction maps generated by digestion of pcel6A.C, pcel6A.BC, pcel6A.BCB, pcel6A.CBC, and pcel6A.CBCB with NdeI and HindIII restriction endonucleases. M: 1Kb DNA ladder.
